# Pulsed electromagnetic field effectively improves musculoskeletal degeneration and inflammation levels in a rat model of osteosarcopenia: an experimental study

**DOI:** 10.3389/fimmu.2026.1795683

**Published:** 2026-03-27

**Authors:** Qi Song, You-kang Zhu, Hai Liu, Xiao Liu, Yuheng Yang, Xi-fang Liu

**Affiliations:** 1 Honghui Hospital, Xi’an Jiaotong University, Xi’an, China; 2 Guangzhou University of Chinese Medicine, Guangzhou, China

**Keywords:** inflammation, osteoporosis, osteosarcopenia, pulsed electromagnetic fields, sarcopenia

## Abstract

**Background:**

Insufficient protein synthesis significantly contributes to the metabolic imbalance between muscle and bone, serving as a primary factor in the concurrent deterioration of both muscle and bone structure and function in the elderly. Increasing evidences suggest that Pulsed Electromagnetic Fields (PEMF) can enhance the recovery process of bones and muscles as a non-invasive and highly specific intervention, thereby offering potential treatment options for osteoporosis and sarcopenia. However, the application of PEMF in the treatment of Osteosarcopenia (OS) remains relatively underexplored, and the associated mechanisms have yet to be elucidated.

**Methods:**

Ovariectomy was performed on rats followed by intraperitoneal injection of excessive dexamethasone. After successful model establishment confirmed by dual-energy X-ray absorptiometry, rats were subjected to 15 Hz, 2.0 mT PEMF intervention (five times per week for 12 weeks). Following the intervention, muscle mass and strength, bone microarchitecture, bone biomechanical strength, serum markers of osteogenic and myogenic differentiation, systemic inflammatory levels, skeletal muscle and bone tissue histomorphology, and protein expression were quantitatively assessed. These analyses were performed to elucidate the regulatory effects of PEMF on chronic musculoskeletal deterioration and circulating inflammatory levels.

**Results:**

We found that PEMF enhance the synthesis of osteogenic and myogenic proteins, leading to an increase in both the quantity and density of trabecular bone and myotubes. This intervention reduces the progression of bone loss and muscle atrophy while simultaneously improving the biological performance of muscle and bone. Moreover, PEMF treatment attenuated the systemic chronic inflammatory milieu, as evidenced by reduced circulating levels of IL-6 and TNF-α, although a complete restoration to baseline levels was not achieved.

**Conclusion:**

PEMF demonstrates therapeutic effects on OS by promoting the synthesis of key proteins involved in osteogenesis and myogenesis, thereby enhancing muscle function and bone strength, while also suppressing the systemic chronic inflammatory microenvironment. These findings provide experimental evidence supporting PEMF as a potential non-pharmacological intervention strategy for OS.

## Introduction

Osteosarcopenia (OS) is a systemic disorder of the musculoskeletal system characterized by the concurrent occurrence of sarcopenia (SP) and osteoporosis (OP). It manifests as reduced bone mineral density, increased bone fragility, and diminished skeletal muscle mass and strength. Due to its strong association with heightened risks of frailty, mobility impairment, and falls, OS has emerged as a significant public health concern (1–3). Epidemiological data indicate that the global prevalence of OS is 18.5%, with a significantly higher incidence among individuals aged over 80 years (PR = 7.64) (4, 5). Although the precise etiology of OS remains unclear, it is believed that a combination of factors—including genetic predisposition, mechanotransduction, systemic chronic inflammation, and fluctuating hormone levels—contributes to its development (1, 6). Furthermore, the temporal and spatial overlap between SP and OP results in a mortality rate that is nearly double that of patients without these comorbidities. Therefore, managing OS has become a critical objective for enhancing the quality of life in older adults.

Despite the critical situation regarding OS, there is currently a lack of effective clinical protocols that can simultaneously improve both muscle mass and bone density. Existing pharmacological studies have primarily focused on evaluating the potential effects of drugs approved for the treatment of OP in enhancing muscle function. However, the long-term efficacy and clinical safety of these drugs require further validation. Compounding this issue, drug therapy for OP may exacerbate myalgia symptoms, as seen with bisphosphonates, while muscle-enhancing agents, such as selective androgen receptor modulators, may disrupt bone metabolic balance (7–9). This therapeutic contradiction underscores the urgent need for the development of non-pharmacological alternative therapies that promote synergistic effects on musculoskeletal health.

As a non-invasive and highly safe physical factor therapy, although PEMF has demonstrated significant potential in the repair and regeneration of bone and muscle (10, 11), its protective effects on tissue damage are related to the extent of the injury and the parameters of the intervention. This raises questions about the universality of PEMF in rehabilitation applications. Moreover, current studies on the effectiveness of PEMF predominantly focus on single tissue types, while systematic research on OS—a complex pathological model involving the coordinated degeneration of muscle and bone—remains lacking. Therefore, there is a need to establish standardized animal models to evaluate its protective effects on muscle and bone, and to seek universal parameters that can delay or reverse musculoskeletal degeneration.

Chronic inflammation, as a common etiology of age-related diseases, has a bidirectional regulatory relationship with protein metabolism imbalance in the pathogenesis of OS. Driven by the senescence-associated secretory phenotype, an increasing number of cells exit the cell cycle and continue to release inflammation-related cytokines, creating a unique malignant cycle of ‘inflammation-metabolism-functional decline’ (12, 13). These inflammatory factors participate in the expression of Wnt/β-catenin and NF-κB/TNF-α signaling pathways, resulting in the destruction of bone resorption-formation balance and accelerated degradation and muscle fiber type transformation (14–16). However, whether PEMF can exert a synergistic effect on bone and muscle in OS patients by modulating chronic inflammation currently lacks systematic experimental evidence. Therefore, focusing on the regulation of inflammation levels is beneficial for understanding the pathogenesis and treatment of OS.

Based on the aforementioned research landscape, this study established an OS animal model to conduct a comprehensive, multi−dimensional assessment of the effects of specific−parameter PEMF intervention on the microstructure and physiological function of the musculoskeletal system as well as systemic inflammatory levels. Furthermore, it sought to elucidate the role and potential underlying mechanisms through which PEMF mediates dual improvement in muscle and bone metabolism.

## Materials and methods

### Experimental design and surgical procedures

Female Sprague-Dawley (SD) rats (8 weeks old, weighing 200–250 g) were obtained from the Laboratory Animal Center of Xi’an Jiaotong University (license No. SCXK-2023-002). Following an acclimatization period until 12 weeks of age, the rats were randomly assigned to three groups (n = 8 per group): the control group (Con), the osteosarcopenia model group (OS), and the pulsed electromagnetic field group (PEMF). Throughout the animal experiments, randomization was employed, and the investigators were blinded to the group assignments during data collection and analysis. All experimental procedures, including animal modeling, were approved by the Scientific Research Ethics Committee of Xi’an University of Physical Education (approval No. XAIPE2023050) and were performed in strict compliance with the animal care guidelines of the Animal Breeding Room of Xi’an Red Scroll Hospital Affiliated with Xi’an Jiaotong University.

**Figure 1:**
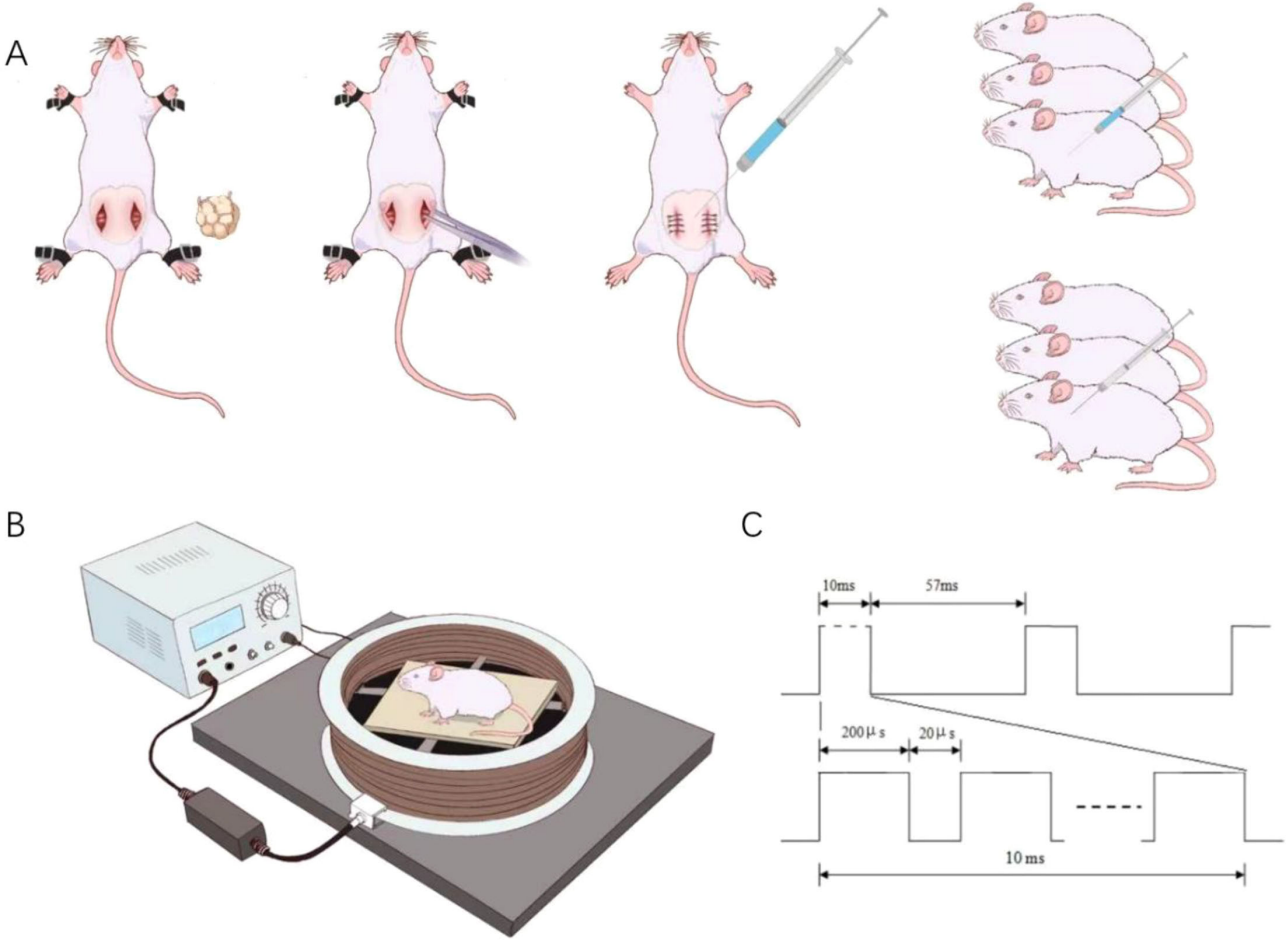
**(A)** Schematic diagram of modeling. **(B)** Intervention mode diagram of pulsed electromagnetic field. **(C)** Pulsed Electromagnetic Field waveform pattern diagram.

Given that estrogen deficiency and excessive glucocorticoid exposure are established etiological factors for both SP and OP, an OS rat model was developed in this study by combining ovariectomy with intraperitoneal dexamethasone administration, following a well-established modeling approach based on this pathophysiological rationale (17). The detailed modeling procedure was as follows: Rats were weighed and anesthetized via an intraperitoneal injection of pentobarbital sodium (40 mg/kg). For the Con group, a comparable mass of peri-ovarian adipose tissue was excised without removing the ovaries. Rats in the OS and PEMF groups underwent bilateral ovariectomy. Following layer-by-layer suture closure, all rats received intramuscular injections of penicillin (40,000 U/kg) once daily for 3 consecutive days to prevent postoperative infections. Pharmacological interventions commenced two weeks post-surgery. Rats in the OS and PEMF groups received intraperitoneal injections of dexamethasone sodium phosphate (2.5 mg/kg), while those in the Con group received an equivalent volume of normal saline. This regimen was administered five days per week for 12 consecutive weeks. One week after model establishment, body weight and forelimb grip strength were measured, and the relative grip strength was calculated for all rats. Subsequently, muscle mass and femoral bone mineral density (BMD) were assessed using dual-energy X-ray absorptiometry (DXA). The data revealed statistically significant reductions in BMD, muscle mass, and muscle strength (P < 0.05) compared to control values, confirming the successful induction of the OS model. (The specific modeling procedure is shown in **Figure 1A**).

### Pulsed electromagnetic fields exposure

The PEMF coil was mounted on a wooden platform and secured using insulating materials. During the intervention, each rat was positioned at the center of the coil, and the intervention process is controlled by the switch of the control instrument. The magnetic field intensity at the coil center was measured using a gaussmeter. The setup was placed in a quiet room maintained at 21–25 °C, free from metal objects and other electromagnetic interference. Based on previous studies from our group and others, as well as considerations for clinical translation, PEMF parameters were set at 15 Hz and 2 mT (18, 19). Rats received PEMF exposure for 30 min per session, 5 days per week. Rats in the Con group were subjected to an identical placement procedure within the coil, but the PEMF device was not activated. The entire intervention period spanned 12 weeks. (The specific procedure and parameter waveforms of pulsed electromagnetic field intervention are shown in **Figures 1B, C**).

### Relative grip measurement

The grip strength of the rats was assessed using a digital grip strength meter. Each rat was gently lifted by the tail, allowing its forelimbs to naturally grasp the probe of the dynamometer while maintaining a horizontal body position. The tail was then pulled back horizontally at a uniform speed until the rat released its grip. The maximum peak force (in Newtons, N) displayed was recorded. This measurement was repeated three times per animal, and the average of these trials was calculated as the absolute grip strength. Subsequently, the relative grip strength was determined using the following formula: relative grip strength (N/kg) = absolute grip peak (N)/body weight (kg).

### Dual-energy X-ray inspection

After modeling and intervention, the body composition of the rats were quantified using DXA. Following anesthesia, the animals were positioned in the scanning chamber of the DXA instrument for a whole-body scan. Region of interest analysis was subsequently performed to determine specific parameters, including femoral BMD and muscle mass.

### Femur three-point flexion test

The three-point bending test on rat femurs was performed using a CMT4304 electronic universal testing machine. Following an axial preload of 2 N to eliminate gaps, a vertical load was applied at a constant displacement rate of 2 mm/min until complete fracture occurred. The support span was set to 8 mm. Key biomechanical parameters, including the modulus of elasticity, yield strength, maximum bearing capacity, and fracture stress, were derived from the resulting stress-strain and force-displacement curves.

### Micro-CT scanning

The left femur was fixed in 4% paraformaldehyde and scanned using a micro-CT system (SkyScan 1276, Bruker, Germany) at an isotropic resolution of 4 μm. A ROI for trabecular bone analysis was defined within the distal femoral metaphysis. This ROI commenced 2 mm proximal to the growth plate and extended a further 1.6 mm (encompassing 400 consecutive tomographic slices). Bone microarchitecture within this volume was quantified using CTAn software (Bruker), which provided measurements of bone volume fraction (BV/TV), BMD, structure model index (SMI), and trabecular number (Tb.N).

### Enzyme-linked immunosorbent assay

Serum levels of the bone formation marker P1NP, the bone resorption marker CTX-1, the myogenic factors IGF-1 and myostatin, and the inflammatory cytokines IL-6 and TNF-α were determined using ELISA kits.(#RA20172, #RA20168, #RA20613, #ERA00039, #RA20607 and #RA20035,Bioswamp, Wuhan, China).

### Histomorphometric analysis

The femoral and gastrocnemius muscle samples were fixed in 4% paraformaldehyde for 48 hours, thoroughly rinsed with phosphate-buffered saline, and decalcified in 10% ethylenediaminetetraacetic acid at 4 °C for 21 days. Subsequently, the samples were embedded in paraffin and horizontally sectioned at 5 μm thickness. The sections were stained with hematoxylin and eosin (H&E) for histological examination of trabecular bone morphology, osteocyte lacunae, and the arrangement of myotubes in the gastrocnemius muscle.

### Western blotting

Total protein was extracted from tissues or cells using RIPA lysis buffer (Bioworld Technology, #AC21191, St. Louis Park, MN, USA). Following quantification, protein samples (30 µg per lane) were separated by SDS-polyacrylamide gel electrophoresis (SDS-PAGE) on 8%, 10%, or 12% gels and subsequently electrotransferred onto polyvinylidene fluoride (PVDF) membranes (Pierce, #24585, Shanghai, China). The membranes were blocked with 5% low-fat milk for 2 hours at room temperature and then incubated overnight at 4 °C with specific primary antibodies. The primary antibodies used were as follows: RUNX-2 (1:1000), Osteocalcin (1:1000), FNDC5 (1:2500), Myogenin (1:750), and GAPDH (1:10000)(#AF5186, #DF12303, #DF13019,#DF8273,Affinity,Jiangsu,China). After incubation with the primary antibodies, the membranes were probed with a horseradish peroxidase (HRP)-conjugated goat anti-rabbit secondary antibody (1:10000) for 1 hour at room temperature. Protein bands were visualized using an enhanced chemiluminescence (ECL) detection system (GE Image Quant 350, GE Healthcare, Fairfield, CT, USA), and the band intensity was quantified by measuring the grayscale values using ImageJ software (National Institutes of Health, USA).

### Statistical analysis

Statistical analyses were performed using SPSS Statistics (version 27.0; IBM Corp., Chicago, IL, USA). Continuous data are presented as means ± standard deviation. For comparisons among three or more groups, one-way analysis of variance (ANOVA) was employed. When measurements were taken at multiple time points within the same subjects, repeated-measures ANOVA was applied to assess changes over time. The P-value of less than 0.05 was considered statistically significant. All graphical representations of the data were created using OriginPro software (version 2024; OriginLab Corporation, Northampton, MA, USA). Representative experiments, such as micrography, were repeated at least two or three times, and all data were derived from our experimental results.

## Results

### Successful establishment of OS rat models

**Figure 2:**
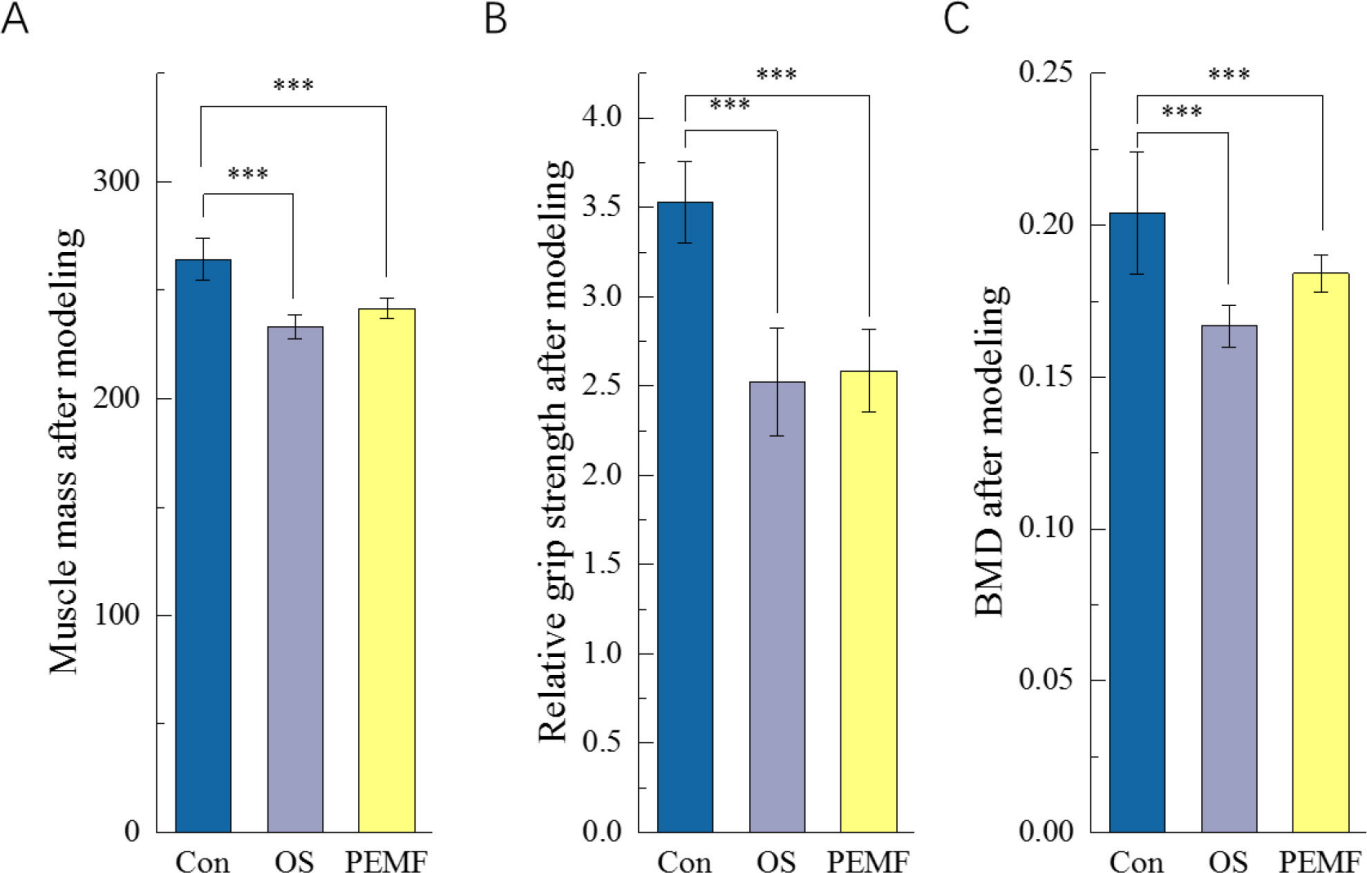
Criteria for determining successful model establishment after 12 weeks of modeling. **(A)** Comparison of muscle mass content in rats after modeling. **(B)** Differences in relative forelimb grip strength results among groups after modeling. **(C)** Femoral bone density results of rats in each group after modeling. “***” indicates a statistically significant difference between the two indicated groups, with p<0.001.

An OS rat model was established in this study through bilateral ovariectomy to induce estrogen deficiency, combined with intraperitoneal injections of dexamethasone to simulate glucocorticoid excess. As illustrated in **Figure 2**, rats in both the OS and PEMFs groups showed significant decreases in muscle mass and femoral BMD from Con group following the modeling procedure. Total body lean mass and femoral BMD were reduced by an average of 34% and approximately 40%, respectively, which aligns with the fundamental pathological characteristics of OS. At the functional level, a marked decrease in relative grip strength was also observed (**Figure 2B**), providing further evidence of the synchronized decline in musculoskeletal integrity. The concerted deterioration of these key metrics confirms the successful establishment of the OS animal model, which is therefore suitable for subsequent investigations into underlying mechanisms or therapeutic interventions.

### PEMF attenuates muscle loss in OS rats by regulating muscle protein synthesis and degradation

**Figure 3:**
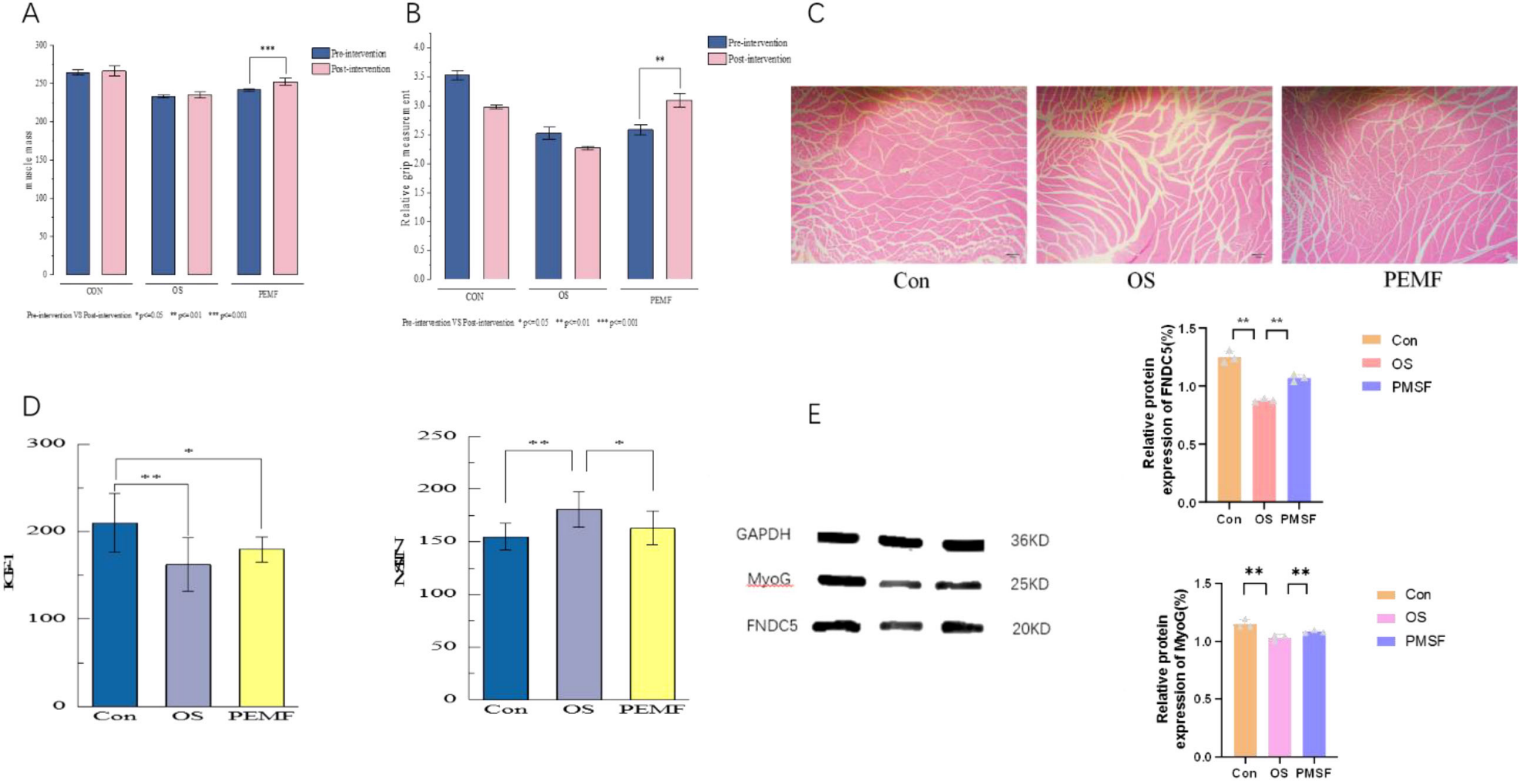
Validation of PEMF on muscle structure and function in different groups of rats. **(A)** Differences in muscle mass content among rat groups in Dual-energy X-ray results. **(B)** Comparison of relative forelimb grip strength among different rat groups. **(C)** Typical HE staining results of gastrocnemius muscle in each group of rats. **(D)** ELISA results showing the levels of circulating IGF-1 and MNST in the three groups. **(E)** Western blot analysis of myogenic protein levels (FNDC5, MyoG) in the gastrocnemius muscle. “*”, “**” and “***” indicate statistically significant differences between the indicated groups, with p<0.05, p<0.01, and p<0.001 respectively.

To assess the effects of PEMFs intervention on muscle mass, structure, and molecular markers in OS rats, we performed DXA, grip strength tests, H&E staining, ELISA, and WB. The results demonstrated that PEMF intervention significantly ameliorated the decline in muscle mass, strength, and tissue architecture compared to the OS group. Specifically, in DXA scans revealed a significantly higher total body muscle mass in the PEMF group compared to the OS group (**Figure 3A**). Furthermore, grip strength tests indicated that PEMF intervention effectively enhanced the functional muscle strength of the rats (**Figure 3B**). Histological analysis by H&E staining demonstrated that PEMF intervention led to increased cross-sectional area of gastrocnemius muscle fibers, more regular and tightly packed arrangement, and a marked reduction in atrophic features, returning the muscle morphology to a state close to that of the Con group (**Figure 3C**). At the molecular level, ELISA of serum samples revealed a significant increase in the concentration of Insulin like Growth Factor (IGF-1), a key promoter of protein synthesis, and a significant decrease in the concentration of Myostatin (MSTN), a negative regulator of muscle growth, in the PEMF group compared to the OS group (**Figure 3D**). Notably, WB analysis of gastrocnemius muscle tissue showed that the protein expression levels of Fibronectin Type III Domain Containing 5 (FNDC5) and the myogenic regulatory factor Myogenin (MyoG) were significantly upregulated in the PEMF group relative to the OS group, albeit still lower than those in the Con group (**Figure 3E**). Collectively, these multi-faceted results demonstrate that PEMF intervention effectively counteracted muscle loss and dysfunction in OS rats by enhancing muscle mass, improving grip strength, restoring normal muscle fiber morphology, and promoting the expression of key anabolic molecules.

### PEMF reduces bone loss in OS rats by regulating trabecular bone quantity and density

**Figure 4:**
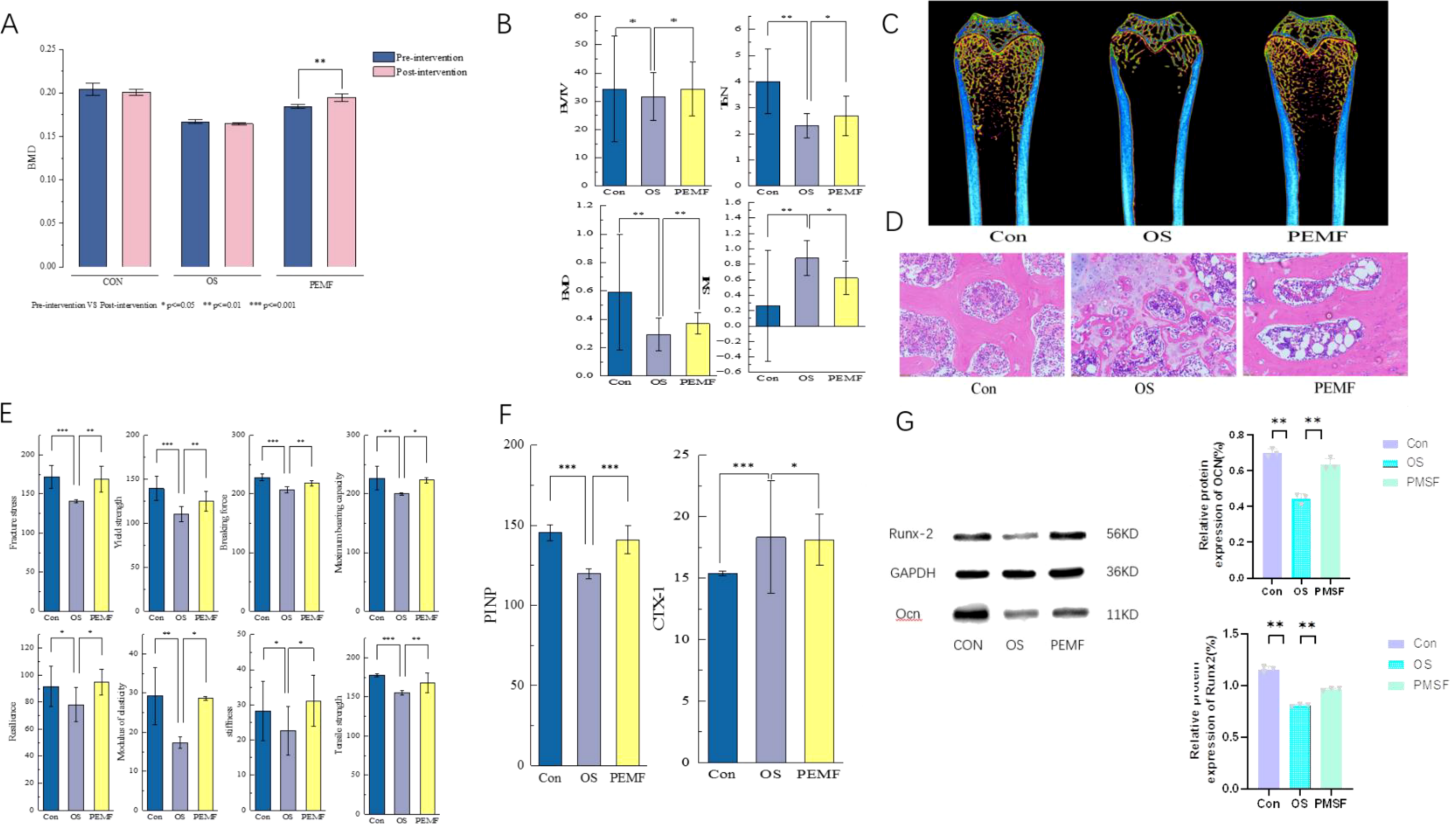
**(A)** Selection of femoral region bone mineral density results in Dual-energy X-ray absorptiometry. **(B)** Comparison of micro-CT trabecular bone scanning results in femurs. **(C)** Representative images of three-dimensional micro-CT reconstruction of femurs in each group of rats. **(D)** Typical HE staining results of femoral bones in each group of rats. **(E)** Comparison of femoral mechanical properties in three-point bending test of femur. **(F)** ELISA results showing the levels of circulating PINP and CTX-1 in the three groups. **(G)** Western blot analysis of osteogenic protein levels (OCN, Runx-2) in different groups of rats. “*”, “**” and “"***” indicate statistically significant differences between the indicated groups, with p<0.05, p<0.01, and p<0.001 respectively.

Subsequently, a systematic analysis was conducted on bone characteristics of OS rats subjected to PEMF intervention to evaluate bone microstructure, biomechanical properties, and molecular expression. Micro-CT analysis revealed that, compared to the OS model group, 12-week PEMF treatment significantly increased the trabecular bone volume and improved the spatial architecture of the bone microstructure. Quantitatively, the BV/TV and BMD increased by approximately 15%, the Tb.N increased by 6%, and the SMI decreased by 26% (**Figure 4B**). These changes suggest a transition of the trabecular bone from a rod-like to a plate-like morphology, indicating enhanced mechanical stability. Consistent with the Micro-CT findings, DXA scans confirmed a significant increase in whole-body bone mass following PEMF intervention (**Figure 4A**). Biomechanical evaluation using the three-point bending test demonstrated a significant increase in the femoral elastic modulus (approximately 53%) in the PEMF group. Furthermore, parameters including yield strength, fracture stress, and toughness increased by 17–27%, while stiffness and tensile strength improved by 6–11% (**Figure 4E**). These results indicate that PEMF intervention effectively enhanced the bone’s resistance to deformation and fracture. Histological examination revealed a significant increase in the number and density of tibial trabeculae, along with a more dense and organized arrangement of collagen fibers, in the PEMF group compared to the OS group (**Figure 4D**). At the molecular level, ELISA revealed a significant increase in the serum concentration of the bone formation marker Procollagen I N - terminal Propeptide (P1NP) and a significant decrease in the bone resorption marker C-terminal Telopeptide of type I collagen (CTX-1) in the PEMF group (**Figure 4F**). WB analysis further indicated that the protein expression levels of Runt-related transcription factor 2 (Runx-2) and Osteocalcin (OCN) in femoral tissue were significantly upregulated in the PEMF group relative to the OS group, albeit remaining lower than those in the Con group (**Figure 4G**). Taken together, PEMF intervention delayed key pathological features of OS by improving bone microstructure, enhancing biomechanical strength, and rebalancing bone metabolism toward formation over resorption, ultimately protecting skeletal integrity.

### Sensitivity of serum inflammatory factor concentrations to PEMF reflection

**Figure 5:**
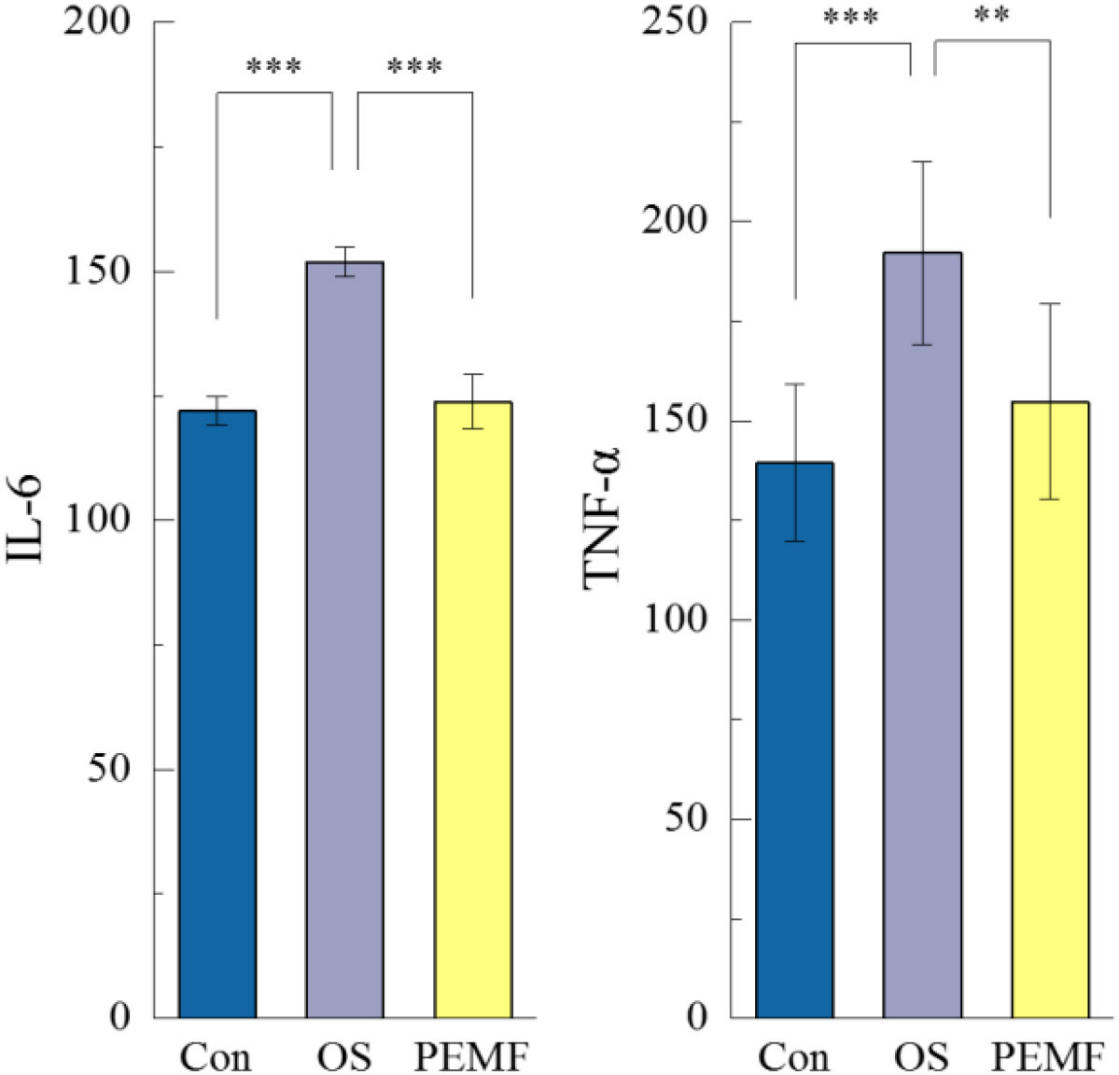
ELISA results showing the levels of circulating IL-6 and TNF-α in the three groups. “**” and “***” indicate statistically significant differences between the indicated groups, with p<0.01 and p<0.001 respectively.

Serum levels of Interleukin-6 (IL-6) and Tumor Necrosis Factor-alpha (TNF-α) were measured to evaluate systemic inflammation in OS rats. The results indicated that estrogen deficiency combined with chronic glucocorticoid exposure significantly elevated the levels of IL-6 and TNF-α, consistent with a state of systemic inflammation. However, PEMF intervention significantly reduced serum concentrations of IL-6 and TNF-α (P<0.01, P<0.001), but did not completely recover (**Figure 5**). This demonstrates that PEMF intervention effectively suppresses inflammation and ameliorates the pro-inflammatory state​ induced by estrogen deficiency and chronic glucocorticoid exposure.

## Discussion

Previous studies have indicated that estrogen deficiency and excessive use of glucocorticoids can lead to a decline in the quality and function of bones and muscles, as well as induce chronic inflammation in the body. This condition is particularly prone to the onset of OS in the elderly population. However, the underlying mechanisms have not been elucidated (20, 21). To counteract this, PEMF has recently been selected as a non-invasive treatment method for restoring musculoskeletal function. However, its therapeutic potential on OS, a condition characterized by the comorbid decline of bone and muscle, remains underexplored. This study aimed to evaluate the protective effects of PEMF on the musculoskeletal system in a rat model of OS and to elucidate the underlying potential mechanisms. The successfully established OS rat model demonstrated significant musculoskeletal degeneration compared to the Con group, exhibiting reductions in muscle mass, grip strength, and BMD, along with a deterioration of bone microstructure. Following a 12-week PEMF intervention (15 Hz, 2 mT), treated OS rats showed a significant attenuation of chronic inflammation. This was associated with upregulated expression of osteogenic and myogenic marker proteins, improved trabecular bone architecture, increased muscle fiber size, and enhanced biomechanical properties of bone and muscle function. These findings suggest that PEMF represents a promising, non-invasive, and cost-effective strategy for mitigating aging- or drug-induced musculoskeletal deterioration.

OS as a comorbidity of SP and OP cross-linking, most intuitively manifested by a decrease in skeletal muscle mass and strength and a reduction in bone density. For skeletal muscle, it is embodied in the change in the arrangement of muscle fibers, accompanied by changes in muscle mass and strength. When physical inactivity or external injury such as denervation occurs, the balance of protein synthesis and decomposition within skeletal muscle is disrupted, and the volume of muscle fibers decreases accompanied by a decrease in contractility and fatigue resistance. Repair of this damage requires myoblasts to differentiate and fuse into myotubes, eventually developing into a complete fibroblast. PEMF plays an important role in this process. In this study, it was observed that after 12 weeks of PEMF stimulation in OS rats, the total muscle mass and relative grip strength of forelimbs were significantly increased compared with those in the OS group, indicating that PEMF could improve the motor function of rats in terms of muscle mass and muscle strength. Torretta’s research also confirms this viewpoint: continuous PEMF stimulation can regulate the synthesis of slow muscle fiber myosin and force transmission proteins in the skeletal muscles of rats with muscle damage, ultimately restoring muscle contraction function (22). Clinical trials have also demonstrated the benefits of PEMF: targeted interventions on the quadriceps and gastrocnemius muscles of SP patients showed that PEMF therapy significantly improved lower limb muscle strength and functional activity in elderly individuals with SP (23). This enhancement in muscle function is also observed in populations without muscle atrophy. One study indicated that PEMF intervention in sedentary youth not only enhanced warm-up readiness​ but also increased muscle response amplitude, resulting in elevated oxygen consumption and energy release (24). Therefore, PEMF’s enhancement of muscle motor function is not limited to sarcopenic populations but has a broad promoting effect on muscle function recovery.

In our further observations of the microstructure of the gastrocnemius muscle in rats, we found that after intervention, the degree of myocyte fusion increased, with indistinct cell boundaries, gradually restoring the complete muscle fiber structure. This is consistent with the findings of Xu, indicating that pulsed electromagnetic fields can improve the myocyte differentiation process, thereby promoting the transition of muscle fiber types (25). Previous studies have demonstrated that therapeutic magnetic stimulation increases the diameter of type I, IIA, and IIB muscle fibers, a change attributed primarily to improved intramuscular circulation and enhanced cellular metabolic activity. This mechanism may likewise contribute to the effects of PEMF at the microscopic level (26). In addition, we observed significant upregulation of both FNDC5 and the myogenic factor MyoG in muscle tissue, suggesting that PEMF promotes the secretion of myogenic proteins and thereby enhances the biological function of skeletal muscle.

Circulating levels of IGF-1 typically decline with age. Upon stimulation, skeletal muscle secretes IGF-1, which subsequently activates the AKT/mTOR signaling pathway, thereby promoting protein synthesis (27). In contrast, MSTN, a member of the transforming growth factor-beta superfamily, is recognized as a key negative regulator of skeletal muscle growth. It functions primarily by activating the SMAD 2/3 pathway, which induces cell cycle arrest in myoblasts, thereby inhibiting their differentiation and fusion into myotubes. Furthermore, MSTN suppresses the activation and proliferation of satellite cells, ultimately impairing muscle regeneration capacity following injury (28, 29). In the present study, PEMF treatment significantly increased the serum level of IGF-1 while concurrently decreasing MSTN, which was consistent with our expectations and matched fluctuations in bone metabolism markers. Therefore, we can affirm that PEMF can enhance the structure and function of muscle fibers by regulating the secretion of various myogenic factors, ultimately having a positive significance for the recovery of motor function in the body. However, the intrinsic mechanisms underlying these changes have not been clearly elucidated in current research, indicating a need for further studies to provide evidence.

Bone loss is a hallmark manifestation of OS, characterized by reduced BMD and deterioration of bone microstructure. Therefore, quantitative assessment is essential for evaluating bone health. Our results demonstrated that the combination of glucocorticoid excess and estrogen deficiency significantly decreased BMD and impaired both bone microstructure and biomechanical properties in OS model rats. In response to these insults, PEMF intervention delayed the progression of bone loss, increased trabecular bone density, and maintained key biomechanical parameters—such as maximal femoral load and flexural strength—at levels superior to those in the untreated OS group. These findings align with recent work by An and Ding, who observed similar outcomes following PEMF intervention in models of postmenopausal or glucocorticoid-induced OP, and which were linked to elevated BMP-2 protein expression *in vivo* (30, 31). This fully demonstrates the restorative effects of PEMF on bone structure and biomechanical properties. Furthermore, this specific protective effect is not limited to female animals; it also exhibits unique bone-protective effects in castrated male osteoporotic rats. Specifically, PEMF partially restores bone formation and reduces bone resorption without affecting iron metabolism, effectively delaying lumbar vertebral bone loss (32). Serum bone metabolism markers are the gold standard for quantitative assessment of bone remodeling processes. We used CTX-1 and PINP, recommended by the International Osteoporosis Foundation, as specific indicators to measure bone metabolism in OS rats (33). Our research found that PEMF enhances the level of bone formation in rats while inhibiting the rate of bone resorption, thereby increasing the differentiation of bone marrow mesenchymal stem cells into osteoblasts and the mineralization level of osteoblasts. Meanwhile, we observed that PEMF significantly upregulates the expression of bone metabolism-related genes OCN and RUNX2. This is because electromagnetic signals can promote the proliferation and differentiation of osteoblasts by altering the amplitude of intracellular calcium transients induced by extracellular high calcium stimulation (34). Previous studies have also demonstrated that PEMF can act on osteoclasts by increasing the apoptosis rate of osteoclasts through the activation of voltage-gated calcium channels, thereby reducing the number of osteoclasts and decreasing their activity. This results in an improvement in bone metabolism levels, thus exerting an anti-osteoporotic effect (35). These studies provide evidence for our understanding of how PEMF can reverse bone loss and enhance bone structure and biomechanical properties.

Chronic low-grade inflammation is recognized as a common pathological basis for various age-related diseases, where the accumulation of pro-inflammatory cytokines amplifies systemic oxidative stress and accelerates disease progression (36). The elderly population uses glucocorticoids to control inflammatory diseases; however, inappropriate application of glucocorticoids often leads to skeletal muscle atrophy and glucocorticoid-induced osteoporosis. Additionally, the decline in estrogen levels can increase the probability of developing these conditions and upregulate the expression of inflammatory factors in the body (37, 38). Therefore, inflammatory cytokines such as IL-6 and TNF-α have emerged as promising biomarkers for the screening, diagnosis, and therapeutic evaluation of both OP and SP (39). Recent research indicates that PEMF not only directly contributes to the restoration of bone and skeletal muscle structure and function, but also regulates the metabolic activity of the local microenvironment through the modulation of systemic inflammation. In the present study, we observed a significant increase in serum levels of IL-6 and TNF-α in the OS rat model compared with the Con group, which correlated with the degree of musculoskeletal degeneration. Estrogen deficiency is known to mildly activate the immune system, accelerating protein degradation, impairing muscle fibrosis, and disrupting bone remodeling balance, ultimately contributing to the development of OS (40, 41). Following 12 weeks of PEMF intervention, the serum concentrations of IL-6 and TNF-α were significantly reduced in the treated group compared with the OS model group, indicating an anti-inflammatory effect of PEMF. Previous studies have demonstrated that inflammation signaling pathways such as NF-KB and STAT3 play a crucial role in the development of osteoporosis and skeletal muscle injury, serving as key genes linking OP and SP (42–45). In rat models of OP and osteoarthritis, PEMF have been shown to inhibit the expression of NF-KB/RANK proteins by activating Sirt1, thereby counteracting the progression of osteoarthritis and delaying the deterioration of bone density and biomechanical properties (46, 47). Furthermore, human trials have also found that PEMF can accelerate fracture healing and reduce tissue inflammation, with its effects on cartilage degeneration and osteoarthritis being validated (48). Thus, the reduction in serum IL-6 and TNF-α levels demonstrates the suppressive effect of the magnetic field on chronic inflammation, consequently preventing the progressive degradation of skeletal and muscular tissues. Interestingly, our findings reveal a dissociation between systemic inflammation and musculoskeletal recovery. Specifically, although PEMF normalized serum IL-6 and TNF-α levels, the structure and function of the musculoskeletal were not fully restored. This discrepancy may be explained by two non−mutually exclusive mechanisms: first, inflammatory pathways might respond more rapidly to PEMF modulation than the processes underlying structural repair; second, although the 12−week intervention was sufficient to suppress inflammation, it may have been inadequate to fully reverse accumulated muscle atrophy and bone loss in this established model. This observation aligns with the clinical reality that arresting disease progression is often more readily achieved than restoring lost tissue mass and function. Thus, our study confirms the anti−inflammatory and partial tissue−reparative effects of PEMF. Notably, to date no clinical studies have investigated the ability of PEMF to mitigate chronic inflammation in patients with OS or SP, underscoring the exploratory nature of our work and highlighting the need for future clinical validation.

Meanwhile., the neurovascular components associated with musculoskeletal tissues play a critical role in tissue homeostasis and repair. Existing evidence indicates that peripheral nerves regulate motor function not only through electrical signal transduction but also via neurosecretion and neuro−tissue interactions, directly participating in the metabolic regulation and structural repair of muscle and bone (49). Concurrently, vascular density and oxygen partial pressure can influence energy metabolism in muscle fibers and bone remodeling. Notably, PEMF has been shown to promote the repair of both nerves and blood vessels (50–52). Thus, we hypothesize that PEMF may synergistically improve the metabolic state of musculoskeletal cells by augmenting neurovascular supply, thereby accelerating the structural and functional recovery of bone and muscle tissues. Overall, these findings underscore the therapeutic value of PEMF in reducing inflammation and enhancing musculoskeletal repair, which presents a novel therapeutic approach for combating OS.

Our study still has certain limitations. First, the observed therapeutic effects are partial rather than curative. The PEMF regimen effectively suppresses systemic inflammation but fails to fully restore musculoskeletal quality and strength to baseline levels. This underscores the necessity for future research to optimize treatment parameters or explore combined strategies involving nutritional or pharmacological interventions to achieve comprehensive functional recovery. Secondly, the clinical efficacy of PEMF for patients with clinical OS needs further validation. Although the intermittent PEMF exposure regimen used in this study is more aligned with clinical application scenarios, our experimental results are based on rodent models rather than the OS population. Therefore, individual variability’s impact on the results cannot be ruled out, which is also a target for our future work. Finally, while we have attempted to select cutting-edge research findings from recent years for the references cited in this article, we did not conduct a strict evaluation of their quality and bias risk, which may also affect the horizontal comparison of research results.

## Conclusion

In summary, this study demonstrates the therapeutic potential of PEMF in alleviating the progression of OS by promoting the repair of musculoskeletal injuries and enhancing locomotor system function. Mechanistically, these beneficial effects are mediated through PEMF’s ability to attenuate systemic inflammation, enhance anabolic processes, and improve the microstructural organization of bone and muscle tissue, thereby mitigating the progression of OS. Collectively, our findings indicate that PEMF represents a promising non-pharmacological strategy, offering a potential adjunctive or alternative therapeutic avenue for the management of OS.

## Data Availability

The original contributions presented in the study are included in the article/supplementary material. Further inquiries can be directed to the corresponding author.
